# The “Ice Age” of Anatomy and Obstetrics: Hand and Eye in the Promotion of Frozen Sections around 1900

**DOI:** 10.1353/bhm.2016.0101

**Published:** 2016

**Authors:** Salim Al-Gailani

**Keywords:** frozen sections, anatomy, obstetrics, visual aids, representation

## Abstract

In the late nineteenth century anatomists claimed a new technique—slicing frozen corpses into sections—translated the three-dimensional complexity of the human body into flat, visually striking, and unprecedentedly accurate images. Traditionally hostile to visual aids, elite anatomists controversially claimed frozen sections had replaced dissection as the “true anatomy.” Some obstetricians adopted frozen sectioning to challenge anatomists’ authority and reform how clinicians made and used pictures. To explain the successes and failures of the technique, this article reconstructs the debates through which practitioners learned to make and interpret, to promote or denigrate frozen sections in teaching and research. Focusing on Britain, the author shows that attempts to introduce frozen sectioning into anatomy and obstetrics shaped and were shaped by negotiations over the epistemological standing of hand and eye in medicine.

In March 1870, anatomist Wilhelm Braune received at his Leipzig institute the body of a young woman who had hanged herself in the final month of pregnancy. Rather than dissect the cadaver, Braune used a still unconventional method. He froze the woman’s corpse solid then sliced it vertically in half to produce the first “frozen sections” of a pregnant body[Other P-611]

**Figure 1:**
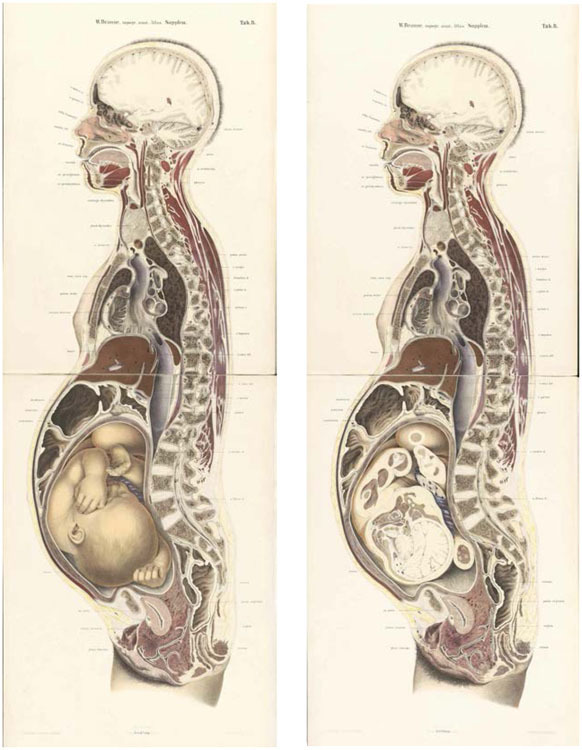
Wilhelm Braune, *The Position of the Uterus and Foetus at the End of Pregnancy. Illustrated by Sections through Frozen Bodies* (Leipzig, 1872). Having sawn the frozen cadaver in two along its middle line, Braune chiseled away the in-utero fetus from the left half to leave the uterine cavity empty. He reunited the fetal parts on the right half of the cadaver to create a trompe l’oeil effect in the final plate. While the sectioned maternal tissue is represented in two dimensions, the lifelike fetus appears in three. These color lithographs (58 x 39 cm) by artist C. Schmiedel appeared in a supplement to Braune’s folio atlas, published simultaneously in English and German.

[Other P-612]

(Figure 1).^1^ Working under a light, Braune traced the outlines of the tissues and cavities on a sheet of transparent paper placed over the frozen surface of the cut section. For “such an elaborate mechanism as the human body,” he explained, “every line must be true to nature and copied with the greatest care.”^2^ Drawings of the tracings, vividly rendered as folio-size color lithographs, were reproduced, debated, and emulated in dozens of publications during what one participant’s history called the “Ice Age” of anatomy and obstetrics between around 1870 and 1910.^3^ Negotiations over the “truthfulness” of this representational technique during this period offer a reach seam of commentary on the purpose of anatomy and its relevance to clinical medicine.

Long praised for their visual sophistication, Braune’s plates are part of the modern canon of anatomical illustration.^4^ Recently, they have been claimed as ancestors of the digitally photographed cryosections that compose the “Visible Human” datasets launched in the mid-1990s and even computerized tomography (CT) scans now in routine clinical use.^5^ More serious assessments have attributed frozen sections to a post-Enlightenment tradition of unflinching realism that produced “stylistically sober” portrayals of the body analogous to an architectural drawing or technical plan.^6^ It is perhaps also tempting to see frozen sections as representatives of a wider regime of objectivity in scientific images around 1900. In the nineteenth century, Lorraine Daston and Peter Galison have argued, an ideal of representation that permitted manipulation in order to depict types was [Other P-613] challenged by a “mechanical objectivity” committed to self-restraint and the unadorned depiction of individuals.^7^ These interpretations capture something important, but are either too general to explain the arguments for and against frozen sections during their heyday or too focused on Braune’s plates to account for the fate of the technique in others’ hands.

Taken up at institutes across Europe, the British Empire, and North America around 1900, frozen sectioning was poised between dissection and the many strategies used to produce “visual displays” for teaching and research.^8^ Nineteenth-century anatomy professors considered dissection essential for inculcating in students the observational skills and manual dexterity required for surgery. While illustrations, models, and preparations were often disparaged as works of art rather than science, these were nevertheless ubiquitous pedagogical tools. Such emerging specialties as histology, pathology, and embryology worked around the growing dominance of dissection to carve out new roles for visual aids designed to communicate knowledge of objects that were especially small, rare, complex, or transient. Anatomists, artists, and technicians experimented with a bewildering array of techniques for rendering bodies and body parts for classrooms, museums, and publications.^9^ With so many methods to choose from, why adopt frozen sectioning?

Enthusiasts credited the technique with no less than revolutionizing understanding of “topography” of the body. This was because frozen sections offered special advantages in demonstrating complex spatial relationships and were allegedly more faithful to living anatomy than existing methods of study. But some medical practitioners doubted that frozen sections were either truthful or useful, maintaining that anatomical knowledge flowed only from direct engagement with bodies, dead and alive. To understand these positions, we need to go beyond comparing isolated pictures to recover the changing social relations of their production and use. In assessments of frozen sections, widely available attitudes [Other P-614] to faithful representation were refracted through disciplinary agendas and struggles over resources.

This essay concentrates on the reception of frozen sectioning in Britain, where the politics of the technique, bound up in little researched deliberations over the status of anatomy and the usefulness of dissection, are especially clear. Histories of medical education around 1900 give the impression that the ascendant laboratory sciences rendered anatomy virtually irrelevant within the preclinical curriculum, while writing on corpse supply to medicine has tended to treat the subject as synonymous with dissection.^10^ Yet frozen sectioning arrived in Britain on a wave of investment in new facilities for anatomical instruction in the late nineteenth century, and cadavers were still much in demand for research, including within clinical disciplines such as surgery and obstetrics. For although dissection is usually seen as the sum total of anatomical practice, negotiations over frozen sections demonstrate that clinicians continued to participate in the field and sought to shape the methods through which it was studied and taught.^11^ After outlining general claims made for and against frozen sections, I trace their use in British anatomy and obstetrics as research tools and teaching devices and show that anatomists’ and clinicians’ disputes over the technique hinged on divergent commitments to tactile and visual knowledge.^12^ Reconstructing these debates over the epistemological standing of hand and eye both helps to grasp what was at stake for advocates and critics and expands our understanding of the place of anatomy in medicine around 1900.

## The “Freezing Method”

Frozen sections came to prominence in Germany within one of the many specialized research programs to emerge from the breakup of anatomy in the mid-nineteenth century. This was “topographical” anatomy, which [Other P-615] analyzed the body regionally rather than by organ system or tissue type, as was more conventional. Though disparaged by practitioners of morphology, the study of organic form, as too descriptive and insufficiently scientific, topographical anatomy claimed greater relevance to the practical needs of the clinic.^13^

By the 1880s several of the larger German universities accommodated the anatomical orientations favored by clinicians and those preferred by preclinical scientists by dividing teaching between two chairs. At Leipzig the former military surgeon Wilhelm Braune taught human topographical anatomy and Wilhelm His comparative anatomy, embryology, and histology. Both professors practiced a “mechanical” anatomy allied to the physicalist physiology of their colleague Carl Ludwig. They argued that the form of the human body could not be understood apart from function, nor development without mechanical principles.^14^ Braune helped to unite topographical and physiological orientations by using sections to research the mechanics of stance and gait.^15^

Braune systematized a method of cutting sections from frozen corpses rather than the usual specimens preserved for weeks in spirits. A few nineteenth-century anatomists had experimented with frozen sectioning, but the technique was little known until Braune’s *Topographisch-anatomischer Atlas* (1867–72).^16^ This was part of a wider Central European trend of importing resources from analytical mechanics, the physical sciences, and industry to develop new techniques of manipulating, visualizing, and preserving anatomical structures.^17^ Traditionally, anatomical preparation making required skill and experience, and “preparators” could guard [Other P-616] their methods jealously.^18^ Frozen sectioning was in theory “open to all,” or at least to those with access to large institutes and research material.^19^ In the 1880s, the technique was confined to the top medical schools and few students would have encountered a frozen section directly. By 1900, however, the “freezing method” was practiced across Europe, North America, and the British Empire.

**Figure 2:**
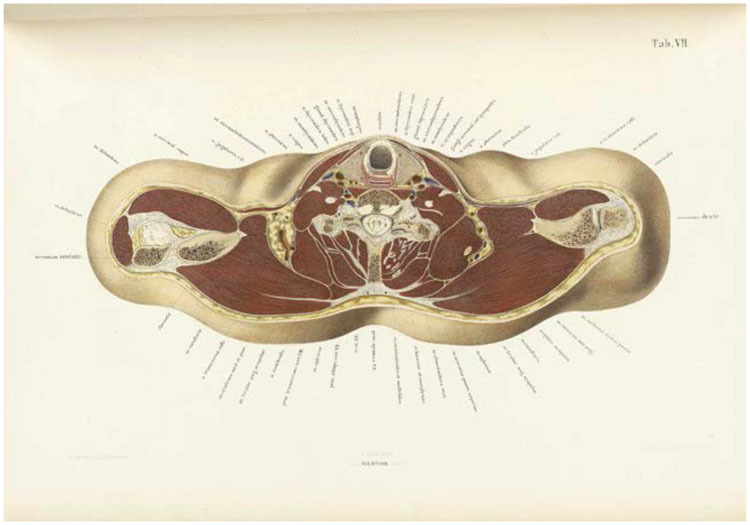
Transverse section cut just below the larynx, from Braune’s *Topographisch-anatomischer Atlas* (Leipzig, 1872; plate VII), color lithograph (56 × 39 cm) by C. Schmiedel. Braune cut the original section from the cadaver of a twenty-one–year-old man.

Braune packed cadavers in a watertight metal case, placed in a large tank and surrounded with two parts chipped ice or snow to one part salt. Widely used before electrical refrigeration, saline freezing mixtures brought the temperature inside a vessel to as low as minus [Other P-617] twenty-one Celsius.^20^ Minus eighteen degrees was reckoned enough to freeze an adult human corpse solid, preserving soft, pliable organs in situ within around five days.^21^ The frozen cadaver was then sliced along various planes with a fine-toothed saw. Compared to the sophisticated apparatus used from the 1870s to cut serial sections of microscopic specimens, the method seems crude, but it took skill to slice sections uniformly without disturbing the internal viscera or tearing tissue as it thawed under the blade.^22^ Transverse sections (cut at right angles to the long axis of the body) (Figure 2) were around an inch thick, full-length sagittal sections (cut along the long axis of the body from front to back) thicker still. Practitioners either drew the sections while the tissue remained frozen, or allowed them to thaw and harden in alcohol. Thawed sections could be preserved in spirits and mounted for museum display.^23^

Braune promoted frozen sections as faithfully preserved living tissue. He and other adherents drew on animal experiments showing that the vital functions of vertebrates could survive freezing and thawing and on histological knowledge that “sections of a perfectly fresh tissue which has been frozen will most closely represent living structure.” Freezing was the most “truthful” method of making microscopic or anatomical preparations, allegedly keeping structures intact and leaving colors unadulterated by such fixing agents as chromic acid.^24^

Enthusiasts also claimed that the method allowed anatomists quickly and easily to translate the three-dimensional complexity of the body into two-dimensional representations. Braune’s technique of tracing onto a sheet of wax paper or glass placed directly on the frozen surface of the [Other P-618] section seemed to guarantee an almost effortless accuracy.^25^ “Few students of anatomy attain sufficient command over pen, pencil and brush to be able to make rapid satisfactory freehand drawings,” but even those with no artistic skill could rapidly trace the outlines of organs from the flat surface of a freshly cut section.^26^ Yet Braune relied on artists to turn his tracings into vivid lithographs, providing samples of fresh tissue to make “the drawing-in of the parts . . . clear and correct.”^27^ His representations were explicitly manipulated and idealized to mimic life. A few practitioners photographed the sections directly, but, like Braune, most had artists convert their drawings into lithographs for publication.^28^ Photography provided neither the clarity nor the color of labeled drawings.^29^ So while commitment to accuracy was paramount, faithfulness to the original object did not imply a ban on human intervention. The advantage was rather that the technique imposed continuity on representational routines and placed the burden on the anatomist’s work rather than the preparator’s skill or the artist’s impression. Because the technique eliminated the need to draw anatomical structures in perspective, both cut sections and drawings could be interpreted in two dimensions and were therefore directly comparable.

Allegedly the “most . . . popular textbook of topographical anatomy in Germany” by the late 1870s, Braune’s atlas stimulated much work using frozen sectioning.^30^ The other leading exponents were also teachers of human topographical anatomy who pursued a mechanical approach to the body in their research. Some practitioners followed Braune by sectioning whole adult cadavers; others focused on infants and children or such complex regions of the body as head, thorax, or pelvis. By the 1880s, wood engravings, lithographs, and photogravures of frozen sections circulated internationally in the leading textbooks, not only of human anatomy, [Other P-619] but also increasingly of clinical disciplines and veterinary medicine.^31^ Anatomists exchanged practical knowledge of the technique at professional societies, and meticulously described and debated frozen sections in research publications.^32^ Recognizing that these were the preparations “the student and the practitioner most desire[d] to see,” museum conservators improved methods for mounting frozen sections for permanent display.^33^ As the technique became more widely known, however, claims about the advantages of frozen sections were subjected to greater scrutiny.

## Fallacy in Frozen Sectioning

Judgments about the value of the freezing method hinged on two related issues: the relative merits of frozen sections compared to dissection and their usefulness to clinicians. Nineteenth-century medical educators venerated dissection as essential preparation for practice. They considered dissecting room experience necessary for forming professional character, cultivating manual dexterity and teaching students to recognize complex structures. So it was a bold move for topographical anatomists to claim frozen sectioning as an advance on “old-fashioned” “scalpel and forceps” dissecting. More “faithful” anatomy, they insisted, would do “the clinician a real service” by encouraging better diagnoses and safer surgery.^34^

Adherents asserted that frozen sections compared favorably with “ordinary methods of dissecting, in the determination of the exact position and relations of the viscera and other structures.” Braune’s technique had “dispelled” “many erroneous ideas.”^35^ One proponent compared viewing a frozen section to seeing a jellyfish in its natural habitat: “the living embodiment of symmetry and beauty propelling itself through the clear blue water”; a corpse dissected in the “old-fashioned” manner, by contrast, was like a jellyfish “half embedded in the sand, a shapeless and repulsive mass.”^36^[Other P-620]

Such arguments provoked three criticisms. The first came typically from research anatomists who accepted the freezing method as valuable in principle, but had reservations about deductions from single frozen sections. Against those who treated sections like architectural plans from which viewers would derive “mental pictures” of the living body, these critics argued that the sections should be seen as a means to an end, rather than finished products.^37^ Anatomists should work through serial sections to produce graphic reconstructions of particular organ systems or regions of the body, or model individual organs in plaster or wood.^38^ From the mid-1890s, formalin challenged freezing as a preservative that hardened corpses so they held their shape during cutting and mounting and could be kept indefinitely. Sections of a formalin-fixed cadaver could be dismantled and individual organs reassembled in “the fashion of a child’s model house.”^39^ But many anatomists continued for some years to use both methods.^40^

A second critique suggested that frozen sections, however accurate, tended to confuse the uninitiated. Even enthusiasts who reckoned these “anatomical puzzles” became more intelligible through familiarity conceded that viewers approached them with “bewilderment.”^41^ Unsurprisingly, then, many teachers of anatomy regarded frozen sections as beyond the capabilities of most students.^42^ Detractors argued that since frozen sections depicted only (potentially abnormal) individuals, they could not compete with the collective knowledge built up through centuries of “scalpel and forceps” dissection and examining living subjects.^43^

The third, and most fundamental, criticism dismissed as fallacious the claim that freezing kept the organs and viscera in the positions they [Other P-621] assumed during life. Detractors, typically clinicians who wanted anatomical knowledge from cadavers subservient to bedside experience, argued to the contrary that the freezing process altered the size, proportion, and relations of parts: “the turgor of life,” one suggested, “is very different from the shrunken condition of the corpse.”^44^ Many agreed that frozen sections by Braune and others gave a misleading impression of the shape, position, and texture of parts in the living body, making all tissues appear alike and conveying nothing of their elasticity.^45^ Jena gynecologist Otto Küstner greeted an anatomist’s intervention with evidence from frozen sections in a long-running debate over the normal position of the uterus with the stroppy retort, “we poor gynecologists with our touching fingers groping around in the darkness of inexactness, look up reverently and expectantly to the mother of all medical science, anatomy.” All too often, the gynecologist complained, “anatomical investigation . . . leads us astray.”^46^

These arguments indicate the variety of objections to the freezing method promoted by topographical anatomists. Some researchers found it sufficient only when using sections to reconstruct 3-D views. Many clinicians dismissed frozen sections as misleading or of limited applicability in teaching. For a better grasp of these positions and the interests behind them, the rest of this essay focuses on the prominent debate over frozen sections in Britain.

## The Varieties of British Anatomy, ca. 1900

Frozen sectioning arrived in Britain amid growing debate over the purpose and relevance of anatomy. Late nineteenth-century medical schools began to set new standards for preclinical education by emphasizing laboratory training, especially in physiology and pathology. Meanwhile, teachers in clinical subjects pressed for a greater share of the curriculum to be given to practical bedside instruction. These pressures forced anatomists [Other P-622] to justify the continued significance of their discipline.^47^ Complaints about run-down dissecting rooms with insufficient cadavers, unenthusiastic teaching, and inadequate examinations reinforced concerns that anatomy was in crisis.^48^

Late nineteenth-century anatomists saw themselves as either clinicians or career academics. The dominant approach was “surgical anatomy,” an orientation for which the London hospital schools were renowned.^49^ Most London anatomy teachers lectured part-time but derived most of their income and status from surgery. Research was “quite a voluntary effort” for part-time instructors who identified as surgeons, and had “little time to keep themselves abreast of the advancing state of the science.”^50^ They viewed anatomy as a stepping-stone to a more prestigious post in clinical medicine, teaching anatomy as the “handmaiden of surgery” and dissection as the practical foundation of a surgical career.^51^ The leading textbook, *Gray’s Anatomy*, exemplified this utilitarian orientation, drawing a direct line between the illustrations, the experience of dissection, and the practice of surgery.^52^

By around 1870, a few anatomists had begun to distinguish themselves from the clinicians who dominated teaching of the subject. The expansion of provincial schools, together with the growth of preclinical education at the Scottish universities and later Oxford and Cambridge, created opportunities for teachers identifying themselves as career academics.^53^ Concerned that territory lost to physiology and other experimental [Other P-623] disciplines had left anatomy departments sterile, this group insisted that the subject should be a science in its own right. These self-styled “scientific” anatomists found models for reform in German universities, where full-time professors advanced their field with original research.^54^ From university-based medical schools outside London, scientific anatomists sought to define their field as the “highest branch of biology,” emphasizing embryology, histology, and comparative anatomy as the pillars of instruction in morphology.^55^

Some scientific anatomists disparaged surgical approaches as diminishing their discipline, but most dared not push this argument too far lest it detach “pure” anatomy from students’ requirements altogether.^56^ As physiology and other laboratory-based subjects jostled for space in increasingly congested curricula, anatomists fought to preserve their status as custodians of preclinical medicine. Many among the new generation of academic anatomists were keen to secure their discipline’s identity as a science without sacrificing its clinical relevance. Committed to introducing better remuneration for full-time academic work, teachers were also concerned to revitalize anatomy as a research subject for both professional anatomists and practicing clinicians.^57^ By the 1890s, there was broad agreement that anatomical instruction should be more closely aligned with practitioners’ needs. But rather than return to traditional surgical approaches, these younger teachers sought to expand the repertoire of anatomical pedagogy beyond dissection, emphasizing instead the value of small-group demonstrations, independent study, and practical training in research techniques.^58^ Many departments also began to provide facilities for clinicians to participate in anatomical research and offered new courses in German-style topographical anatomy—sometimes termed “regional” or “applied” anatomy—for advanced undergraduates and post-graduates. [Other P-624] How did this shifting disciplinary landscape shape the British reception of frozen sectioning?

## Between “Fashionable” Preparations and “the True Anatomy”

In 1879, the Royal College of Surgeons of England had a British student of Braune’s make frozen sections for display at the Hunterian Museum in London to “supplement” its already “splendid collection.” The consensus was soon that “every good teaching museum should have its *repertoire* of such specimens.”^59^ As medical institutions both in and outside London invested in new facilities for anatomical teaching at the end of the century, space and resources were set aside for these most “fashionable” preparations.^60^ The variety and quality of preparations had long been signs of an anatomical school’s vitality and an important means of attracting students.^61^ Schools now highlighted their collections of frozen sections as an indication that their anatomical department had been improved for teaching purposes.^62^ Despite broad agreement that frozen sectioning was a valuable new resource, surgical and scientific anatomists differed about what those purposes should be.

These fissures were first apparent in Edinburgh, where anatomists took a leading role in establishing the new technique in the 1880s. The major rival to London as Britain’s foremost center of medical education, Edinburgh was the largest and most prestigious of the Scottish universities.^63^ In William Turner, the university also had the country’s preeminent scientific anatomist. A full-time professor since 1867, he was committed to anatomy teaching in the German style, from the morphological as well as the surgical point of view, by career academics involved in research. With new facilities for anatomical instruction prioritized within a broader program [Other P-625] of institutional redevelopment during the 1870s and 1880s, Turner looked on German universities, especially Leipzig, as models for rebuilding his department. Combined with systematic lectures and dissecting classes, small group demonstrations in human topographical anatomy were a cornerstone of teaching at Edinburgh from the mid-1870s.^64^

Impressed by Braune’s innovations in frozen sectioning, Turner made the technique central to the activity of his refurbished department, opened in 1880.^65^ Generations of demonstrators recruited to assist with teaching were encouraged to regard frozen sectioning as a means of advancing knowledge in human anatomy. The most famous of these, Daniel Cunningham, who succeeded Turner as professor in 1905, used frozen sections to reconstruct wooden models of the “true forms” of the human stomach, kidney, liver, and spleen.^66^ Equipping practicing clinicians with “more precise” anatomical knowledge, Turner and his protégés argued, would give “greater definition” to their diagnoses and interventions.^67^

Produced in abundance for research, frozen sections also gained an important role in the department’s teaching. In practical anatomy classes, students typically encountered dismembered, often decayed corpses, fulfilling the requirement to dissect an entire body part by part, often over several years.^68^ Then there was the shortage of cadavers, a perennial source of concern for anatomy instructors in Edinburgh; in 1880, 650 students shared a meager eighty-five bodies.^69^ These constraints, the teachers [Other P-626] alleged, made it difficult to visualize the whole, living body. Studying the sectioned frozen cadavers displayed in the university’s “magnificent” new dissecting hall was “necessary to correct . . . impressions gained by dissection,” which “artificially” separated parts and “disturbed” relations.”^70^ Students learned to make reconstruction drawings of organs from serial sections, and where resources allowed, the more advanced were encouraged to produce their own. Through sustained engagement with sections a pupil “gradually builds up in his own mind a picture of the human body,” considered impossible through piecemeal dissection alone. At the university, then, studying frozen sections was part of the “mental training in exact observation” Turner considered the key to a higher anatomy.^71^

Like many Scottish anatomists, Turner contrasted his approach with what he portrayed as the narrowly “professional” training on offer in London. But even as he built up the university department as a bastion of anatomical science, a second group of teachers in Edinburgh cultivated a parallel pedagogical tradition that resembled the London model. Existing alongside the university was an “extramural” medical school, which taught for the diplomas of the corporate licensing bodies, the Royal Colleges of Physicians and of Surgeons. A long-standing source of competition to the professoriate, these independent teachers attracted students by targeting perceived weaknesses in the university curriculum.^72^ In the 1880s, many still claimed that anatomy could be effectively taught only in relation to practical surgery.^73^ Especially dangerous for Turner’s department were the courses in surgical anatomy run by the senior clinical staff of the Edinburgh Royal Infirmary, who had first access to “teaching material.” Seeking to exploit student grievances about the limited opportunities in the city for hands-on experience, these courses promised anatomical instruction tied closely to clinical pathology and operative surgery. For these teachers, [Other P-627] students should “view and study anatomy through surgical spectacles” either at the bedside on living patients, or in the postmortem room.^74^

In judging frozen sections, these surgically oriented teachers drew on long-standing assumptions about the role visual aids should play in anatomical pedagogy. While preparations, plates, and models were pervasive in classrooms, generations of educators had denied that they could ever rival dissection.^75^ Although preparations were better established and more tolerated than either drawings or models, the dominant view among surgeons was that visual aids all too often distracted students from gaining direct experience handling and observing corpses. Because surgery was a “handicraft,” teachers argued, “costly apparatus, splendid cabinets, magnificent plates” were mere diversions, useless for building students’ confidence with knife and forceps.^76^

Surgeons by and large tolerated frozen sections as preparations so long as they were used “merely for reference or for the completion of the anatomical series.”^77^ One of Edinburgh’s leading surgeons, James Spence, thus accepted in 1881 that frozen sections were “useful aids in their proper place,” but denied they had any bearing on the cultivation of the “clinical method,” “skilful . . . diagnosis and treatment of surgical disease.” Spence was typical in balancing praise for frozen sections as museum preparations with the warning that, like any visual aid, they were no substitutes for “actual careful dissection of the body, the student working *for himself*.”^78^ More generally, where drawings of frozen sections appeared in textbooks of surgical anatomy, they were always secondary to schematic diagrams that taught general principles, or representations that depicted the body as it would be seen in the dissecting room or operating theater.^79^[Other P-628]

Surgeons viewed frozen sections as just one variety of visual aid among many. At the university, by contrast, the technique represented a novel way of studying human anatomy that exposed the didactic shortcomings of dissection. In what would become the leading English-language dissecting guide, Cunningham went so far as to write that “sectional anatomy is the true anatomy.” It was among Edinburgh-trained anatomists of Cunningham’s generation that frozen sectioning would take hold. The technique offered them an attractive prospect: a “promising field of research” and a new orientation in anatomical instruction that was clinically relevant and yet distinct from the established surgical tradition.^80^ By the mid-1890s, the university had formalized this aspect of training by instituting a new lectureship in “regional anatomy.”^81^ Anatomists justified the emphasis on frozen sectioning by pointing to the “extended range of modern surgery as a consequence of anaesthesia and antisepsis.” More interventionist, complex surgery, they argued, required a more accurate knowledge of the positions and relations of organs than dissection alone could provide.^82^

By now Edinburgh-trained anatomists were filling new full-time professorial chairs elsewhere. Twenty-three of Turner’s former students went on to hold appointments at universities across the British Empire and North America, where many would promote the gospel that frozen sections were indispensable to anatomical teaching and research.^83^ But that is not the whole story. While anatomists were the main users, frozen sectioning also attracted a second group in Edinburgh.

## Reforming Obstetric Anatomy

Traditionally subordinate to medicine and surgery, obstetrics with gynecology had become prominent in nineteenth-century medical education. Courses in these subjects had substantial anatomical components; teachers had long claimed that advanced knowledge of anatomy, transmitted through formal training, distinguished the (male) “science of obstetrics” [Other P-629] from the (female) “art of midwifery.”^84^ Nineteenth-century obstetricians judged Scottish-born man-midwife William Hunter’s 1774 *Anatomy of the Human Gravid Uterus* an unsurpassed “masterpiece . . . of careful dissection and accurate drawing.”^85^ But female cadavers were notoriously scarce and tended to be reserved for anatomical or surgical instruction. Obstetric teachers therefore relied heavily on textbook diagrams, models, plates, and specimens to instruct students in normal female anatomy and the various stages of pregnancy and parturition, together with all manner of diseases and complications.^86^ How did frozen sections inform obstetric approaches to studying anatomy?

Published in 1872 as a supplement to his atlas, Braune’s pregnancy plates alerted “adventurous obstetricians” to the potential of frozen sectioning to refine their understanding of the anatomy of the gravid uterus. The plates had soon “attained wide circulation” in Edinburgh, where obstetrics was unusually well established.^87^

The university had Britain’s oldest midwifery chair, held since 1870 by Alexander Russell Simpson, nephew of previous incumbent and anesthetic pioneer James Young Simpson. An Obstetrical Society, founded in 1839 to promote this emerging specialism as a formal branch of medicine, encouraged both corporate identity and scientific enterprise. Long before frozen sections arrived, obstetricians in Edinburgh were accustomed to associating the collection, preparation, display, and discussion of anatomical and pathological specimens, drawings, and models with the advancement of both individual careers and the standing of their discipline.^88^ The university [Other P-630] justified A. R. Simpson’s appointment on the grounds that the new professor would have “the advantage of possessing his uncle’s invaluable museum, medical library, diagrams, apparatus, and other appurtenances used in lecturing.”^89^ Simpson earned a reputation as a cosmopolitan and progressive instructor, his teaching “filled with the latest obstetric knowledge of the Continent.” If his enthusiasm for frozen-section anatomy was motivated by a concern to improve the university’s teaching apparatus with the latest in German innovations, it was also grounded in a commitment to advancing obstetrics and gynecology with “serious scientific research.”^90^

By around 1880, a handful of European obstetricians and gynecologists were making their own frozen sections, though always fewer than the anatomists who were better positioned to access cadavers.^91^ Inspired by these continental exemplars, Simpson encouraged his best students to seize opportunities to advance obstetric science, rather than merely receiving knowledge from anatomy. At the same time, then, that anatomists in Turner’s department were adopting frozen sectioning, Simpson led a generation of his own protégés in using the technique to cultivate a parallel tradition of obstetric anatomy. A stream of influential studies on the sectional anatomy of the female pelvis and fetus prompted other obstetricians and even anatomists to recognize a distinctive “Edinburgh School” of obstetrics.^92^

Together with Simpson, two students in particular, David Berry Hart and Alexander Hugh Freeland Barbour (Simpson’s son-in-law), made frozen sectioning central to their identities as “scientific obstetricians.” Having adopted the technique while working as Simpson’s teaching assistants in the early 1880s, both moved into extramural lectureships and, soon after, clinical positions at Edinburgh’s major lying-in hospital, the Royal Maternity, and on the gynecological wards of the Royal Infirmary. Members of the senior clinical staff at these institutions were permitted to make use of the bodies of deceased patients for teaching and research. Simpson, Hart, and Barbour succeeded in claiming some of this material for frozen sectioning, including rare corpses of pregnant women who had died in labor. In a body of work spanning around three decades, [Other P-631] they would contend that obstetricians and gynecologists, who seldom had opportunities to dissect, could learn most from scarce resources by embracing sectional anatomy.^93^

Hart and Barbour achieved international recognition with their *Manual of Gynecology* (1882), which carried many drawings of frozen sections and insisted that sectional anatomy should form the very foundations of obstetric and gynecological pedagogy and practice.^94^ In this and other publications, the authors reiterated anatomists’ general arguments for the technique: that frozen sections came “closest to [capturing] the actual condition” of the living body and were the most effective means of studying the “true” positions and relations of parts.^95^ But despite the common ground, there were important differences in emphasis.

**Figure 3:**
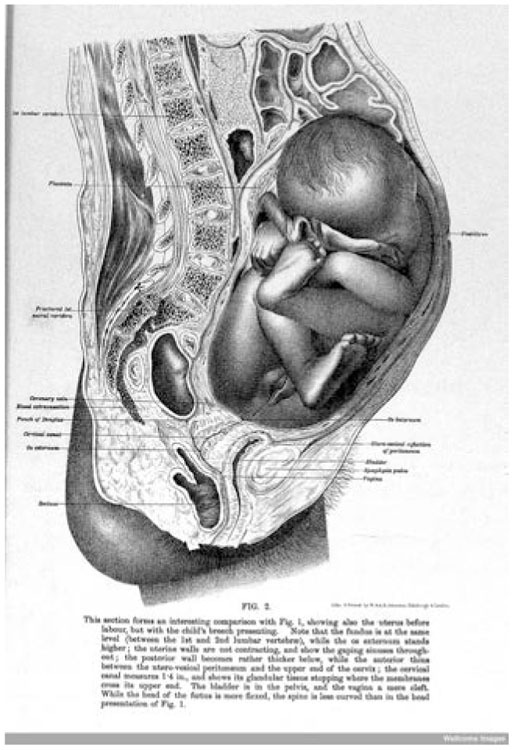
Lithograph of a vertical section of the uterus before labor, from Alexander H. F. Barbour’s *Atlas of the Anatomy of Labour* (Edinburgh, 1889), published in black-and-white, elephant folio size. Note the similarity to Braune’s plate (Figure 1). Wellcome Library, London.

First, obstetricians of the Edinburgh School highlighted the potential of the technique for investigating dynamic physiological and pathological processes beyond static structures. Barbour, for instance, concentrated on the mechanism of labor, comparing sections cut from the cadavers of women who had died at different stages of advanced pregnancy to build “an instructive picture” of the changes in the shape and position of the uterus, fetus, and placenta during childbirth. For Barbour, collecting a “complete series of sections from every stage” would enable obstetricians to “read the changes” as if they were “turning over the pages of a book” (Figure 3).^96^

Second, obstetricians invoked frozen sections to critique the casual use of pictures within their discipline. Unlike anatomists, they were not interested in using sections to reconstruct 3-D views, but engaged with drawings of bodies bisected along a single plane. Representations of the female pelvis in cross section had long been a fixture of obstetric works. [Other P-632] For Edinburgh obstetricians, frozen sections were more authentic than these “diagrammatic” illustrations of medical theories, surgical procedures, and diagnostic techniques. Claiming to show “things exactly as they are,” they contrasted the “*representing* of portions of the body as they might appear in section from the *making* of sections as a means of investigation.”^97^ Barbour explained that with frozen sections,

Nothing is left to the imagination. It is remarkable how much of what we are accustomed to regard as ‘knowledge’ is imaginary; we get one fact, and fill in all the rest from the imagination. We have only to compare any of the numerous diagrams in obstetrical works with an actual section to see how many erroneous ideas creep in with the single fact which the diagram was intended to express.^98^

In other words, obstetricians should not be content with “mere” diagrams, but should inform their teaching and practice with “actual” anatomical research.

[Other P-633]

Third, obstetricians pressed even more strongly than anatomists the claim that “ordinary dissection” tended to mislead, and that frozen sections were required to “eliminate sources of error.” The Edinburgh School “carried the war into the camp” of surgical anatomists, charging that the standard works had long introduced students to female anatomy with diagrams sold misleadingly as “actual representations.”^99^ Hart and Barbour derided the cross sections of the female pelvis in *Gray’s Anatomy*—still the dominant textbook for British medical students—as riddled with errors, and bemoaned the overreliance of “older anatomists” on dissection.^100^ They also alleged that since dissecting room anatomy largely depended on dismembered male cadavers preserved in spirit, neither anatomical teachers nor their students could appreciate the texture and form of the living female pelvis to the same degree as practicing clinicians.^101^ Because so much surgery relied on dissection, medical students were exposed to erroneous ideas about gynecological conditions.^102^

Members of the Edinburgh School thus claimed a larger role not just for obstetrics, but also for gynecology within the medical curriculum. During Simpson’s tenure, the university required degree students to attend a six-month course in midwifery, with one lecture per week on the diseases of women. For a medical license, students needed only three months of midwifery. These constraints meant that a significant portion of gynecological instruction, including in the anatomy of female pelvis, was left to dissecting classes, or general medicine and surgery. Edinburgh obstetricians maintained that this unsystematic training was inadequate. Their work in frozen sectioning both exposed curricular failings and bolstered demands for more gynecological instruction by specialists. The university recognized this need by creating a separate lectureship in gynecology, awarded to Barbour on Simpson’s retirement in 1905.^103^ In his new position, Barbour promoted sectional anatomy as ideal for preclinical teaching. Depicting “relations of parts as they are found in the living subject,” drawings of frozen sections introduced students to the form, size, and [Other P-634] position of normal and pathological structures that they would later learn to recognize at the bedside.^104^

Beyond teaching, however, members of the Edinburgh School sought to establish a lineage for frozen sections as part of an independent tradition of obstetric anatomy going back to Hunter.^105^ These practitioners found encouragement in a medical culture increasingly favorable to research. Obstetricians were among the first clinicians to make use of the new Laboratory of the Royal College of Physicians of Edinburgh, opened in 1887 to provide research facilities and diagnostic services for the local medical elite.^106^ Obstetric advocates of frozen sectioning also benefited from a fund established by the college to subsidize the cost of preparing illustrations for their publications, as well as numerous bequests to the university to foster “original scientific work” by younger members of the profession.^107^ The main setting for these investigations was the Obstetrical Society, which in 1883 convened a special committee to “induce Fellows to preserve for exhibition and examination preparations they might have been ready to throw aside as useless.”^108^

Presenting preparations and drawings of frozen sections also in the Medico-Chirurgical Society, the Pathological Club and the Royal Physical Society allowed a form of “virtual witnessing” of the fruits of the Edinburgh School’s research by the city’s medical elite.^109^ Collective judgments about the credibility of individual reports worked to build consensus that frozen sectioning had inaugurated “a new era in obstetrics,” providing both “anatomical facts” and a new pictorial idiom that should form the “immediate basis of clinical work.”^110^ Under Simpson’s leadership, Hart and Barbour established a set of procedural and representational conventions in obstetric anatomy that subsequent generations would be encouraged, even obliged, to emulate. The frozen section became an emblem of Edinburgh’s obstetric elite.[Other P-635]

## The Digital Eye and the Ocular

Having discussed the arguments anatomists and obstetricians in Edinburgh used to promote frozen sectioning, I now focus on the more critical stances. For although praise for the technique overwhelmed any significant dissent in the Scottish capital, not all medical practitioners accepted frozen sections as the “true anatomy.”

Advocates of frozen sectioning claimed that the new technique promised to put clinical work on a secure “anatomical basis,” with more “correct” views of the body than could be obtained by any other method. In adopting this strategy, Edinburgh anatomists and obstetricians met with skepticism, even hostility, from counterparts elsewhere, and especially in London. Here, elite medical culture differed from that of Edinburgh. Hospital schools, rather than universities, dominated medical education, research was a relative rarity, and specialization was denigrated as fostering narrow-mindedness. Hospital doctors were often distrustful of innovations derived from the preclinical sciences, which they perceived as undermining the physician’s holistic and intuitive clinical skills. On the contrary, diagnostic and therapeutic judgment could be developed only through personal experience of practice itself.^111^

Metropolitan clinicians deployed such rhetoric against sectional anatomy. Operating in a professional world that considered diagnostic acumen a higher virtue than specialist expertise, these physicians and surgeons tended to regard frozen sections as unwelcome infringements of their clinical authority. The default position here was that conclusions drawn from frozen sections “must harmonise with clinical phenomena to be accepted.”^112^ Robert Barnes, one of the country’s best known gynecological surgeons, was typical among London-based clinicians in expressing his disdain for frozen sections. He discouraged treating sections as “trustworthy evidence of the position of pelvic organs.” Neither preparations nor pictures were able to convey “turgescence of the blood-vessels, the play of the muscles, and other vital conditions. . . . Clinical explorations, although seemingly less precise,” he argued, “give really better information.”^113^ For Barnes, the *tactus eruditus*, the educated touch, was the ultimate arbiter [Other P-636] of clinical truth. This could be cultivated, he insisted, only through long practice in “feeling the various conditions of form, size, consistency, and relations of the parts upon which this sense is to be exercised.”^114^ As purely anatomical evidence, frozen sections could therefore never supersede the “clinical method” derived from “unsurpassed experience.”^115^

Such views chimed with a broader ambivalence to frozen sectioning among London-based anatomy teachers. Continuing to see visual aids as either luxury adjuncts or “inadequate substitutes for dissection,” metropolitan surgical anatomists remained unconvinced by claims that frozen sections were essential classroom aids. Charles Lockwood, demonstrator of anatomy and operative surgery at Saint Bartholomew’s Hospital, presented the standard suspicions to an audience at the British Medical Association annual meeting in 1888. Lockwood accepted that such preparations “may in a trifling degree assist in training the powers of observation,” but protested that they did nothing to teach “manual dexterity and the use of instruments, [or] familiarity with the appearance, texture, and consistence of the different tissues.” If the purpose of teaching anatomy was to discipline hand and eye, frozen sections privileged the latter at the expense of the former.^116^

No anatomist would have denied that dissection was the bedrock of medical training. But such criticisms pushed some university-based teachers, asserting themselves in debates about educational reform, to harden their stance. In the most provocative polemic in support of frozen sections yet, Alexander Macalister, recently appointed to the first full-time chair in anatomy at the University of Cambridge, argued in 1893 that there were “limitations of the utility of dissection.” Because dissection rendered the body “like the fallen Humpty-Dumpty,” frozen sections “were the only way of learning relations.” Sections, he insisted, were therefore not “mere superfluities—ornamental adjuncts to a dissecting room [but] necessary parts of the teaching apparatus in any properly equipped school.” They were essential for enabling the practitioner, “without hesitation, to put his finger over or a needle into any structure of the body” and for “mentally picturing how the parts lie with regard to each other.”^117^ A full-time [Other P-637] academic in Turner’s mold, Macalister defined himself as a scientific anatomist, committed to teaching the subject both systematically and topographically.^118^ He insisted that frozen sections and morphology alike invested “the whole subject with an interest which raises anatomy from the level of an engrossing handicraft . . . and makes it a science as well as an art, an exercise of the understanding as well as a training of the senses.” Both were essential elements of the “modern curriculum.”^119^

Macalister incited one London anatomy teacher to open revolt. Thomas Cooke, surgeon to the Westminster Hospital and the proprietor of a long-running private anatomy school, offered supplementary tuition to struggling students and access to corpses during the summer months, when the dissecting rooms of the hospital schools were closed. Several hospital-based anatomy teachers offered private lectures and demonstrations and many “coaches” helped struggling students prepare for exams, but Cooke’s was the only private anatomy school recognized by the medical licensing bodies. Established in 1870, it thrived before being forced from permanent premises when a neighbor sued Cooke for endangering public health by running a dissection room in public view. By 1880, Cooke operated out of a temporary building in a disused Bloomsbury graveyard. Repeatedly threatened with closure, the school nevertheless survived into the twentieth century on a reputation for providing expert tuition to students who wanted more opportunities to dissect.^120^

Institutional insecurity made Cooke an unusually vocal commentator on transformations in anatomical teaching. Lacking prestige and resources, his business depended on student demand for supplementary dissecting and expectations that his courses were identical in content to those at the hospital schools, just better taught and tailored to individual needs. Threatened by a new breed of university-trained anatomist he punned were “divorced by degrees from the humble duties of the consulting room,” Cooke contrasted the “thoroughly honest practical work” of dissecting with the “lofty aspirations” of “scientific anatomy.” An 1893 pamphlet claimed that “scientific anatomists” had been lured “into the clouds of Dreamland” by morphology and the assumed “hyper-correctness” of the frozen section. “Neither [were] guides in operating, nor data verifiable [Other P-638] in dissecting” but led teachers down the “road of plates, diagrams, casts and the like.” Resentful of the superior resources of the universities and hospital schools, Cooke condemned the pretentions of “the favoured few who can keep a freezing tank” for neglecting their main responsibility to help students cultivate a “trained hand” for the clinic. “We should describe what can really and truly be seen by ordinary dissecting room processes as are within every student’s rank, and nothing else,” he wrote of frozen sections; “to the practical mind, scientific truth is conformity to what one learns through one’s own senses.”^121^

Claims that frozen sections were the best means of “learning relations” by Macalister and others drew outrage from Cooke in letters to *Lancet* and an extraordinary attack on what he termed “paper anatomists” in the tenth and eleventh editions of his dissecting guide *Tablets of Anatomy* (1894 and 1898). Cooke insisted that frozen sections were a fad and, like all visual aids, threatened to lead “the student away from the dissecting room, and injure him as a practical man.”^122^ Harking back to “the teaching of the good old time,” Cooke argued that only cultivation of tactile observation by dissection could train surgeons: “the *digital* eye is of more importance than the ‘ocular.’”^123^ Yet “paper anatomy,” he claimed, had pushed “dissecting-room anatomy [into] the background; for speculation is always more attractive than hard work.”^124^ The London surgeon Timothy Holmes, who edited *Gray’s Anatomy* between 1864 and 1880, wrote to *Lancet* to agree with Cooke, who alleged he had the backing of other metropolitan anatomy teachers.^125^ Yet other onlookers saw an outmoded “grinder” whose “denunciations” were “unbecoming.”^126^ Sneering commentaries on the controversy dismissed Cooke as a relic.^127^

Cooke’s struggles bear witness to larger shifts in British anatomy around 1900. Following Edinburgh’s lead, anatomy was coming to be seen as a separate discipline, taught by career academics rather than part-time clinicians. Extramural teaching declined as “university education” was accepted as the standard, and students were expected to further their [Other P-639] training with periods of advanced study and research. Cooke may have been more prepared than other surgical anatomists to proclaim his contempt for frozen sections but was not without support. A *Lancet* editorial endorsed his complaint that dissection was becoming a “lost art,” ironically even as medical schools were expanding facilities for anatomical teaching and acquiring cadavers on an unprecedented scale.^128^ Cooke’s claim that he had the private sympathy of exponents of what he termed the “old schools of anatomy” may not have been so far-fetched.

As the only licensed teacher of dissection in Britain with no affiliation to a medical school, Cooke was exceptional. Yet his campaign against “paper anatomy” brings surgeons’ ambivalence toward frozen sections—and the modernizing project of scientific anatomy—into sharper focus. Academic anatomists singled out frozen sections as necessary teaching apparatus that offered more than the models, plates, and even preparations routinely disparaged as distractions from dissecting room learning. Those who saw anatomy as a “handmaiden to surgery” did not recognize the distinction. For this group, teaching with “hyper-correct” frozen sections was to miss the point of anatomical instruction, to educate surgeons’ fingers rather than anatomists’ eyes. More than a competition for resources, the dispute was a contest over anatomy’s very soul.

## Conclusion

Around 1870 German topographical anatomists systematized a technique that had been known for decades, but only sporadically employed. In Britain, university anatomists adopted frozen sections as exemplary products of German science with which to reassert the relevance of their discipline to research and teaching. This horrified those teachers struggling to keep anatomy solidly in the business of training surgeons and away from the “dreamland” of speculation. Similarly, frozen sections were embraced by academic obstetricians and gynecologists who viewed anatomical research as a means of elevating their discipline, but alarmed those for whom tendentious arguments from pictures challenged the authority of clinical experience. For advocates, handling a corpse or examining a patient no longer sufficed to cultivate an authoritative understanding of the body. Opponents complained that frozen sections privileged ocular over digital expertise.[Other P-640]

In Edinburgh’s academic medical culture, anatomists and obstetricians who embraced frozen sectioning had a common concern with preserving the autonomy of their disciplines, especially from surgery, but used the technique in different ways. For anatomists, learning from frozen sections encouraged the full mental participation that would lead to a higher conception of bodily form and anatomy itself. They defined frozen sections as physical preparations necessary for helping students to cultivate three-dimensional knowledge of normal anatomical relations, and often made reconstructions from series. Anatomists presented the drawing, handling, and contemplation of these preparations as complementing dissection, creating the vivid and permanent “mental pictures” required for accurate diagnosis and surgery. Obstetricians likewise emphasized the tendency of dissection to mislead, but saw the advantage of frozen sections as providing more anatomically correct illustrations of difficult-to-obtain cadavers from which students could learn. Research on frozen sections highlighted the inadequacies of current teaching of female pelvic anatomy, and signaled that students needed more specialist instruction in obstetrics and gynecology.

By the early twentieth century, in part as anatomists and, to a lesser extent, obstetricians trained in Edinburgh took positions at medical schools across the anglophone world, the technique became common elsewhere, too.^129^ Frozen sections gained a surer foothold in teaching as part of a wholesale reevaluation of methods of medical instruction. Reformers encouraged schools to prioritize “inductive” learning, including by expanding opportunities for independent, practical, and often research-based study. In both anatomy and obstetrics, this brought a new appreciation of auxiliary aids, with X-ray and stereoscopic photographs, cinema films, living models, preparations, and frozen or formalin-hardened sections fixtures of practical demonstrations and self-directed learning in laboratories, teaching museums, and study rooms.^130^ Anatomical [Other P-641] departments invested in “freezing apparatus” and even dedicated laboratories to produce frozen sections for teaching and research.^131^ Yet interest in frozen sectioning as a research technique faded in the 1910s, as the diminishing returns of gross anatomy drove advanced students toward the newer laboratory disciplines. Obstetricians recognized that innovation in their field relied increasingly upon bacteriology, experimental pharmacology, and biochemistry rather than anatomy.^132^ Frozen sections continued to be used in undergraduate teaching, but their distinctiveness and appeal perhaps paled when compared to X-rays and as textbooks swelled with ever more various illustrations.

Sectional views of the body are routine in medicine today as clinicians and anatomy teachers exploit computers to visualize internal structures in both two dimensions and three. This has helped revive interest not only in nineteenth-century frozen sectioning as a precursor, but also in the section as a representational device across the sciences and in craft traditions such as architecture and engineering. Sections are claimed to have a unique capacity to convey relations among structures and spatial domains.^133^ The history of frozen sections suggests that we should focus less on their allegedly inherent qualities than on contexts of use. Frozen sections provoked controversy because, around 1900, anatomy and its relations with clinical disciplines were in flux. The arguments recapitulated earlier disputes over the roles of hand and eye in transferring medical knowledge and anticipated more recent ones over modalities of anatomical learning. As cryosections, X-rays, ultrasound, MRI, CT, and 3-D imaging controversially replace dissection in some medical schools, many anatomists still insist upon “hands-on” experience of cadavers.^134^ We should recognize the continuities of these debates, but also appreciate the contingencies that have shaped them.[Other P-642]

## Footnotes

1
BrauneWilhelm, Die Lage des Uterus und Foetus am Ende der Schwangerschaft: nach Durchschnitten an gefrornen Cadavern (Leipzig, 1872). Suicide victims were among the pauper cadavers from which German anatomical institutes drew their supply: BuklijasTatjana, “Cultures of Death and Politics of Corpse Supply: Anatomy in Vienna, 1848–1914,” Bull. Hist. Med. 82 (2008): 570–607.

2
Quoted in EycleshymerAlbertSchoemakerDaniel, A Cross-Section Anatomy (New York: Appleton, 1911), xi.

3
KerrJohn M. MunroJohnstoneRobert W.PhillipsMiles H., Historical Review of British Obstetrics and Gynaecology, 1800–1950 (Edinburgh: E. & S. Livingstone, 1954), 50.

4
ChoulantLudwig, History and Bibliography of Anatomic Illustration in Its Relation to Anatomic Science and the Graphic Arts, new ed., trans. and ed. FrankMortimer (Chicago: University of Chicago Press, 1920), 402; SappolMichael, Dream Anatomy (Bethesda, Md.: U.S. Department of Health and Human Services, National Institutes of Health, National Library of Medicine, 2006), 49–51.

5
RifkinBenjaminAckermanMichael J.FolkenbergJudy, Human Anatomy: Depicting the Body from the Renaissance to Today (London: Thames & Hudson, 2006), 307; and BellingCatherine, “How to Imagine Ourselves,” Lancet 383 (2014): 1711–12.

6
KempMartin, Seen/Unseen: Art, Science, and Intuition from Leonardo to the Hubble Telescope (Oxford: Oxford University Press, 2006), 276; and GuillermeJacquesVérinHélèneSartarelliStephen, “The Archaeology of Section,” Perspecta 25 (1989): 226–57.

7
DastonLorraineGalisonPeter, “The Image of Objectivity,” Representations 40 (1992): 81–128.

8
On visual displays, see BerkowitzCarin, “The Beauty of Anatomy: Visual Displays and Surgical Education in Early-Nineteenth-Century London,” Bull. Hist. Med. 85 (2011): 248–71.2180418510.1353/bhm.2011.0030

9
HopwoodNick, “Artist versus Anatomist, Models Against Dissection: Paul Zeiller of Munich and the Revolution of 1848,” Med. Hist. 51 (2007): 279–308; Hopwood, “Producing Development: The Anatomy of Human Embryos and the Norms of Wilhelm His,” Bull. Hist. Med. 74 (2000): 29–79; AlbertiSamuel, Morbid Curiosities: Medical Museums in Nineteenth-Century Britain (Oxford: Oxford University Press, 2011); JacynaL. S., “‘A Host of Experienced Microscopists’: The Establishment of Histology in Nineteenth-Century Edinburgh,” Bull. Hist. Med. 75 (2001): 225–53; and SchnalkeThomas, Diseases in Wax: The History of the Medical Moulage (Berlin: Quintessence, 1995).

10
On anatomy and the laboratory disciplines in medical education, see BonnerThomas N., Becoming a Physician: Medical Education in Britain, France, Germany and the United States, 1750–1945 (New York: Oxford University Press, 1995), 259–63. For corpse supply, see RichardsonRuth, Death, Dissection and the Destitute (Harmondsworth: Penguin, 1988); and HurrenElizabeth, Dying for Victorian Medicine: English Anatomy and Its Trade in the Dead Poor, c. 1834–1929 (Basingstoke: Palgrave Macmillan, 2012).

11
But see Buklijas, “Cultures of Death” (n. 1) on demand for cadavers beyond anatomy, for practices other than dissection.

12
On earlier debates over hand and eye in anatomy, see BerkowitzCarin, “Charles Bell’s Seeing Hand: Teaching Anatomy to the Senses in Britain, 1750–1840,” Hist. Sci. 52 (2014): 377–400.

13
NyhartLynn K., Biology Takes Form: Animal Morphology and the German Universities, 1800–1900 (Chicago: University of Chicago Press, 1995), 225.

14
Ibid., 80–84; HopwoodNick, “‘Giving Body’ to Embryos: Modeling, Mechanism and the Microtome in Late Nineteenth-Century Anatomy,” Isis 90 (1999): 462–96.1054796610.1086/384412

15
HisWilhem, “Wilhelm Braune,” Archiv für Anatomie und Physiologie (1892): 231–56; and MayerAndreas, Wissenschaft vom Gehen. Die Erforschung der Bewegung im 19. Jahrhundert (Frankfurt: S. Fischer, 2013).

16
BrauneWilhelm, Topographisch-anatomischer Atlas: Nach Durchschnitten an Gefrornen Cadavern (Leipzig, 1867–72); HisWilhelm, “Zur Geschichte der Gefrierschnitte,” Anatomischer Anzeiger 13 (1897): 331–33. For an overview history of sectional anatomy, see EycleshymerSchoemaker, Anatomy (n. 2), ix–xv. Braune’s atlas reproduced drawings of frozen sections from two pioneering midcentury works: PirogovNikolaĭ Ivanovich, Anatome Topographica Sectionibus (Petropoli, 1852–59) and GrandeEugène-Quintien Le, Anatomie Chirurgicale Homalographique (Paris, 1858).

17
WaldeyerWilhelm, “The Relations of Human Anatomy to Other Sciences,” in International Congress of Arts and Science, vol. 9: Biology, ed. RogersHoward (New York: Houghton, Mifflin, 1906), 361–77.

18
PettigrewJ. Bell, “Anatomical-Preparation Making as Devised and Practised at the University of Edinburgh and at the Hunterian Museum of the Royal College of Surgeons of England,” Lancet 158 (1901): 1479–84; BuklijasTatjana, “Public Anatomies in Fin-de-Siècle Vienna,” Med. Stud. 2 (2010): 71–92.

19
BallantyneJohn W., The Diseases and Deformities of the Foetus, vol. 1 (Edinburgh, 1892), 26.

20
Freezing mixtures were explained in public lectures and textbooks on the physical sciences and had long been used in the production of ice cream. For an entry point into this literature, see QuinzioJeri, Of Sugar and Snow: A History of Ice Cream Making (Berkeley: University of California Press, 2009).

21
DwightThomas, Frozen Sections of a Child (New York, 1881), iv.

22
SymingtonJohnson, “On the Preparation and Preservation of Frozen Anatomical Sections,” Proc. Roy. Phys. Soc. Edinburgh 6 (1881): 353–56. Some sections were allegedly “so roughly executed they are practically almost useless”: SymingtonJohnson, The Topographical Anatomy of the Child (Edinburgh, 1887), 41.

23
WilsonJ. T., “On a Method of Mounting and Exhibiting Frozen Sections of the Cadaver in the Anatomical Museum,” J. Anat. Physiol. 45 (1910): 3–6.17232865PMC1288881

24
TaitLawson, “On the Freezing Process for Section-Cutting,” J. Anat. Physiol. 9 (1875): 249–58, quotations on 250. For the history of frozen sectioning in histology as distinct from gross anatomy, see BracegirdleBrian, A History of Microtechnique: The Evolution of the Microtome and the Development of Tissue Preparation (London: Heinemann, 1978), 135; and WrightJamesjr., “The Development of the Frozen Section Technique, the Evolution of Surgical Biopsy, and the Origins of Surgical Pathology,” Bull. Hist. Med. 59 (1985): 295–326.

25
His, “Geschichte” (n. 16).

26
EycleshymerSchoemaker, Anatomy (n. 2), xi.

27
BrauneWilhelm, An Atlas of Topographical Anatomy after Plane Sections of Frozen Bodies, trans. BellamyEdward (London, 1877), 5.

28
BerryRichard, A Clinical Atlas of Sectional and Topographical Anatomy (Edinburgh: Green, 1911); RüdingerNicolaus, Topographisch-chirurgische Anatomie des Menschen, 4 vols. (Stuttgart, 1873–79).

29
On the use of photography in anatomy, see MaehleAndreas-Holger, “The Search for Objective Communication: Medical Photography in the Nineteenth Century,” in Nonverbal Communication in Science Prior to 1900, ed. MazzoliniRenato (Florence: Leo S. Olschki, 1993), 563–86.

30
Lancet 109 (1877): 319.

31
EycleshymerSchoemaker, Anatomy (n. 2), xii.

32
“Professor Rüdinger’s New Sections,” Med. Times Gazette 2 (1879): 564.

33
BillingsJohn S., “Medical Museums, with Special Reference to the Army Medical Museum at Washington,” Boston Med. Surg. J. 119 (1888): 265–273, quotation on 268.

34
DwightThomas, “Demonstration of a Model of the Abdominal Viscera,” Massachusetts Med. Soc. 18 (1899): 741–50.

35
Symington, “Preparation and Preservation” (n. 22), 353 See also DwightThomas, The Anatomy of the Head (Boston, 1876), vi; MacEwenWilliam, Atlas of Head Sections: Fifty-Three Engraved Copperplates of Frozen Sections of the Head (Glasgow, 1893), vii.

36
KeillerWilliam, “The Descriptive Anatomy of the Human Heart,” Proc. Amer. Assoc. Anat. 8 (1896): 85–90, quotation on 85.

37
HenkeWilhelm, Construction der Lage des Herzens in der Leiche aus einer Serie von Horizontalschnitten (Tübingen, 1883), 8–9.

38
Symington, Topographical Anatomy (n. 22); DwightThomas, “Model (4xN) of the Human Abdominal Viscera,” J. Boston Soc. Med. Sci. 2 (1897): 6–7. For reconstructions in embryology, see Hopwood, “‘Giving Body’” (n. 14).PMC212174319971145

39
BirminghamAmbrose, “Some Points in the Anatomy of the Digestive System,” J. Anat. Physiol. 35 (1900): 33–66, quotation on 34. On formalin, see Alberti, Morbid Curiosities (n. 9), 115.17232455PMC1287279

40
Berry, Clinical Atlas (n. 28), 2–3.

41
DwightThomas, “Demonstration of a Model of Frozen Sections of the Arm, with Remarks on This Method of Teaching Anatomy,” Boston Med. Surg. J. 118 (1888): 245–46; “A Discussion on the Relations Which Examinations in Anatomy and Physiology Bear to the Teaching of Those Subjects,” Brit. Med. J. 2 (1890): 679–83, 682.

42
T.J. H., “The Study of Anatomy,” Brit. Med. J. 2 (1893): 1079–80.

43
“Methods of Teaching Anatomy,” Boston Med. Surg. J. 130 (1894): 468; Dublin J. Med. Sci. 104 (1897): 497.

44
RanneyAmbrose L., The Topographical Relations of the Female Pelvic Organs (New York, 1883), 43 See also CoeHenry, “The Anatomy of the Female Pelvic Organs,” in A System of Gynecology, vol. 1, ed. MannMatthew(Philadelphia, 1887), 95–234, quotation on 132; and SymingtonJohnson, “Fallacies in Frozen Sections,” Brit. Med. J. 1 (1888): 44.

45
GoffeJ. Riddle, “Treatment of Retrodisplacements of the Uterus by Shortening the Round Ligaments per Vaginam,” JAMA 31 (1898): 508–14; KellyHoward, “Injuries of the Perineum and Pelvic Floor,” in Mann, System of Gynecology, vol. 2 (n. 44), 719–78.

46
Quotation in HarrisonGeorge T., “Displacements of the Uterus,” in Mann, System of Gynecology, vol. 2 (n. 44), 1092.

47
“The Teaching of Anatomy,” Brit. Med. J. 1 (1874): 350–51; FosterMichael, “The Address in Physiology,” Brit. Med. J. 2 (1880): 309 For comparable debates in the United States, see BlakeJohn, “Anatomy,” in The Education of American Physicians: Historical Essays, ed. NumbersRonald L. (Berkeley: University of California Press, 1980), 29–47; SappolMichael, A Traffic of Dead Bodies: Anatomy and Embodied Social Identity in Nineteenth-Century America (Princeton, N.J.: Princeton University Press, 2002), 313–21; and WarnerJohn HarleyRizzoloLawrence J., “Anatomical Instruction and Training for Professionalism from the 19th to the 21st Centuries,” Clin. Anat. 19 (2006): 403–14.

48
“A Discussion on the Teaching of Anatomy,” Brit. Med. J. 2 (1897): 878–80.

49 “Teaching of Anatomy” (n. 47).

50
Medical Education in London: Being a Guide to the Schools of the University of London in the Faculty of Medicine (London: Ash and Company, 1908), 157.

51
BrownJ. MacDonald, “The Science of Human Anatomy: Its History and Development,” Edinburgh Med. J. 30 (1884): 585–96; quotation on 592; “The International Medical Congress,” Brit. Med. J. 2 (1881): 545–66; FraserAlec, “The Position of Anatomy,” Trans. Roy. Acad. Ireland 12 (1894): 343–55.

52
RichardsonRuth, The Making of Mr. Gray’s Anatomy: Bodies, Books, Fortune, Fame (Oxford: Oxford University Press, 2008).

53
ButlerStella V. F., “A Transformation in Training: The Formation of University Medical Faculties in Manchester, Leeds, and Liverpool, 1870–84,” Med. Hist. 30 (1986): 115–32.351752110.1017/s0025727300045348PMC1139613

54
“Teaching of Anatomy” (n. 47); BanksW. Mitchell, “The Teaching of Anatomy,” Brit. Med. J. 1 (1874): 466–67.

55
ClelandJohn, “Rational Teratology,” Brit. Med. J. 2 (1888): 346–48, quotation on 346. For morphology in British anatomy, see BlackmanHelen, “Anatomy and Embryology in Medical Education at Cambridge University, 1866–1900,” Med. Educ. 40 (2006): 219–26.10.1111/j.1365-2929.2006.02395.x16483324

56
“Teaching of Anatomy” (n. 47), 351 For surgical criticisms of scientific anatomy, see Second Report from the Select Committee of the House of Lords on Metropolitan Hospitals, Mins. of Ev. [paper no. 457] (London: HMSO, 1891), 20, 112.

57
British and Irish anatomy teachers formed a new society to advance these goals in 1887; see “Anatomical Society of Great Britain and Ireland,” J. Anat. Physiol. 21 (1887): 681–82.PMC128870217231720

58
“Discussion on the Teaching of Anatomy” (n. 48); GeddesA. C., “A Scheme for the Teaching of Anatomy,” Brit. Med. J.1 (1912): 1109–12.

59
Quotations from “Frozen Anatomical Sections,” Brit. Med. J. 1 (1879): 203; “Plane Sections of Frozen Bodies at the College of Surgeons,” Lancet 113 (1879): 133.

60
“Glasgow Royal Infirmary Medical School: New Buildings,” Glasgow Med. J. 18 (1882): 450.

61
BerkowitzCarin, “Systems of Display: The Making of Anatomical Knowledge in Enlightenment Britain,” Brit. J. Hist. Sci. 46 (2013): 359–87.

62
See, e.g., “The New Buildings for the Cambridge Medical School,” Brit. Med. J. 1 (1886): 166–67; “The New Medical School: Trinity College, Dublin,” Brit. Med. J. 2 (1887): 907–8; “Hospitals and Medical Schools: Improvements in Buildings,” Brit. Med. J. 2 (1890): 810.

63
LawrenceChristopher, “The Shaping of Things to Come: Scottish Medical Education 1700–1939,” Med. Educ. 40 (2006): 212–18.1648332310.1111/j.1365-2929.2006.02393.x

64
On Turner, see TurnerArthur Logan, Sir William Turner: A Chapter in Medical History (Edinburgh: William Blackwood and Sons, 1919); SturdySteve, “Turner, Sir William (1832–1916),” in Oxford Dictionary of National Biography (Oxford: Oxford University Press, 2004; online ed., 1 2011). On the department’s teaching, see “Anatomical School of Edinburgh,” Brit. Med. J. 2 (1875): 128–29.

65
Turner, Sir William Turner (n. 64), 417; “Anatomical Department of the New Medical School, University of Edinburgh,” Brit. Med. J. 2 (1880): 1032.

66
CunninghamDaniel, “The Form of the Spleen and the Kidneys,” J. Anat. Physiol. 29 (1895): 501–17, quotation on 501. Cunningham left the originals with a carverDublin wood, “in order that he might supply duplicates to those anatomists who might desire to have them” (502).PMC132839817232155

67
TurnerWilliam, “Address at the Opening of the Anatomical Department in the New Buildings of the University of Edinburgh,” Lancet 116 (1880): 724–26; 759–61, quotation on 760.

68
“Anatomical School of Edinburgh” (n. 64); CrowtherM. AnneDupreeMarguerite, Medical Lives in the Age of Surgical Revolution (Cambridge: Cambridge University Press, 2007), 68–69.

69 Inspector of Anatomy Accounts and Reports for 1880, National Archives of Scotland, Edinburgh (Ref. MH2). For complaints by Edinburgh students about the scarcity of cadavers in the 1880s, see *The Student*, December 19, 1887, 43; January 18, 1888, 63.

70
CunninghamDaniel J., Manual of Practical Anatomy (Edinburgh, 1889), vi–vii. On frozen sections in the university dissecting hall, see “Anatomical Department” (n. 65); ShepherdF. J., “Notes of a Visit to Some of the Anatomical Schools and Surgical Clinics of Europe in 1887,” Can. Med. Assoc. J. 14 (1924): 59–65, esp. 63.

71
TurnerWilliam, “On Medical Training and University Organisation,” Brit. Med. J. 2 (1890): 827–30, quotations on 828.10.1136/bmj.2.1554.827PMC220823220753149

72
The literature on extramural medical education is large, but entry points include JacynaL. S., “Theory of Medicine, Science of Life: The Place of Physiology Teaching in the Edinburgh Medical Curriculum, 1790–1870,” in The History of Medical Education in Britain, ed. NuttonVivianPorterRoy (Amsterdam: Rodopi, 1995), 141–52; and Jacyna, “‘A Host of Experienced Microscopists’” (n. 9).

73
See, e.g., BrownMacDonald, “The Science of Human Anatomy” (n. 51).

74
StilesHarold, “Surgical Training: Reminiscences and Suggestions,” Brit. Med. J. 2 (1919): 487–89, quotation of 488; ChieneJohn, “Introductory Lecture on the Past and Future of the School of Medicine of Edinburgh,” Brit. Med. J. 2 (1875): 575–77.10.1136/bmj.2.3068.487PMC234341220769661

75
For Edinburgh, see SecordAnne, “Botany on a Plate: Pleasure and the Power of Pictures in Promoting Early Nineteenth-Century Scientific Knowledge,” Isis 93 (2002): 28–57, esp. 46–47. See also Hopwood, “Artist versus Anatomist” (n. 9); Berkowitz, “Beauty of Anatomy” (n. 8); and MaerkerAnna, “Anatomizing the Trade: Designing and Marketing Anatomical Models as Medical Technologies, ca. 1700–1900,” Tech. Cult. 54 (2013): 531–62.

76
WatsonPatrick Heron, “Introductory Address Delivered at Surgeon’s Hall, Edinburgh,” Edinburgh. Med. J. 12 (1867): 769–93, quotations on 785–86.PMC530836029646493

77 “Plane Sections of Frozen Bodies” (n. 59).

78
SpenceJames, “Inaugural Address Delivered in the Department of Surgery in the New Buildings of the University of Edinburgh,” Med. Times Gaz. 63 (1881): 565–67, quotation on 566–67.

79
Examples include ChieneJohn, Lectures on Surgical Anatomy (Edinburgh, 1878) and TrevesFrederick, Surgical Applied Anatomy, 3rd ed. (London, 1892).

80
Cunningham, Manual of Practical Anatomy (n. 70), vi–vii.

81
The Edinburgh University Calendar 1895–6 (Edinburgh, 1895), 597.

82
BerryRichard J., “Anatomy,” Edinburgh Med. J. 17 (1905): 130–36, quotation on 136. See also Turner, “Address” (n. 67), 761; and RobinsonArthur, “A Glance at Anatomy from 1705–1909,” Edinburgh Med. J. 3 (1909): 405–19.

83
Turner, Sir William Turner (n. 64), 159. Examples of Edinburgh-trained anatomists advocating frozen sections include Symington, Topographical Anatomy (n. 22); Keiller, “Descriptive Anatomy” (n. 36); PrimroseA., “The Study of Anatomy by Frozen Sections,” Can. Practit. 19 (1894): 319–30; and Berry, Clinical Atlas (n. 28).

84
SmithWilliam Tyler, A Manual of Obstetrics, Theoretical and Practical (London, 1858), 1; CunninghamAndrew, The Anatomist Anatomis’d: An Experimental Discipline in Enlightenment Europe (Farnham: Ashgate, 2010), 165–85.

85
BarbourAlexander H. F., Atlas of the Anatomy of Labour (Edinburgh, 1889), 2–3. Studies of Hunter’s atlas include JordanovaLudmilla, “Gender, Generation and Science: William Hunter’s Obstetrical Atlas,” in William Hunter and the Eighteenth-Century Medical World, ed. BynumWilliamPorterRoy (Cambridge: Cambridge University Press, 1985); and MasseyLyle, “Pregnancy and Pathology: Picturing Childbirth in Eighteenth-Century Obstetric Atlases,” Art Bull. 87 (2005): 73–91.

86
We lack a thorough history of nineteenth-century obstetric pedagogy, but see MoscucciOrnella, The Science of Woman: Gynaecology and Gender in England, 1800–1929 (1990; repr., Cambridge: Cambridge University Press, 1993); and NuttallAlison, “Like Uncle, Like Nephew? A Comparison of Lecture Content and Teaching Methods of James Young Simpson and Alexander Russell Simpson,” in James Young Simpson: Lad o Pairts, ed. NuttallAlisonManderRosemary (Erskine: Scottish History Press, 2011), 39–56.

87
Edinburgh Med. J. 18 (1873): 940; and 23 (1877): 69–73, quotation on 73.

88
JenkinsonJacqueline, Scottish Medical Societies, 1731–1939: Their History and Records (Edinburgh: Edinburgh University Press, 1993), xx.

89
“The Town Council Curators on the Recent Midwifery Election at Edinburgh,” Brit. Med. J. 2 (1870): 125.

90
“Sir Alexander Russell Simpson,” Brit. Med. J. 1 (1916): 572–74, quotation on 572; SimpsonAlexander Russell, “The Teaching of Obstetrics,” Brit. Med. J. 2 (1898): 300–303.

91
For a critical survey of this early obstetrical work, see Ranney, Topographical Relations (n. 44).

92
SymingtonJohnson, “Introductory Address,” Lancet 142 (1893): 1107–10; NewmanGeorge, Recent Advances in Medical Education in England (London: HMSO, 1923), 28.

93
See obituaries “David Berry Hart,” Brit. Med. J. 1 (1920): 852–53 and “A. H. Freeland Barbour,” Brit. Med. J. 1 (1927): 1168–69; and McGregorT. N., “The Rise and Development of the Edinburgh School of Obstetrics and Gynaecology, and Its Contribution to British Obstetrics,” Brit. J. Obstet. Gyn. 66 (1959): 998–1006.

94
HartDavid BerryBarbourAlexander H. F., Manual of Gynecology (Edinburgh, 1882). Translated into multiple languages and in its sixth edition by 1904, this became “the standard gynaecological textbook at home, in the Colonies, and in America” around 1900; see Brit. Med. J. 1 (1927): 1168.

95
Barbour, Atlas (n. 85), 8–9; HartDavid Berry, “A Critical Consideration of the Methods of Anatomical Research, with Special Reference to the Topographical Anatomy of the Female Pelvis,” Edinburgh Med. J. 31 (1885): 138–41.PMC548715829613070

96
Barbour, Atlas (n. 85), 11; BarbourAlexander H. F., “Some Recent Results from the Study of Labour by Means of Frozen Sections and Casts,” Brit. Med. J. 2 (1890): 1002–4, 1053–55, quotation on 1055 and 1004.10.1136/bmj.2.1557.1002PMC220836820753161

97
Barbour, Atlas (n. 85), emphasis in original.

98
Barbour, “Some Recent Results” (n. 96), 1002.

99
Trans. Obstet. Soc. Edinburgh 10 (1885): 198.

100 Ibid., 198.

101
HartDavid Berry, “The Nature and Aim of Investigations on the Structural Anatomy of the Female Pelvic Floor,” Trans. Edinburgh Obstet. Soc. 14 (1889): 62–71.PMC548665229612928

102
HartBarbour, Manual (n. 94), 46–7.

103
In 1922, the university reverted to a single professorship in obstetrics and the diseases of women: WatsonBenjamin P., “An Address on the Past, Present, and Future of Midwifery,” Brit. Med. J. 2 (1922): 712–15.2077089010.1136/bmj.2.3225.712PMC2416957

104
BarbourAlexander H. F., “The Teaching of Gynecology,” Edinburgh Med. J. 21 (1917): 274–79, quotations on 277.

105
Barbour, Atlas (n. 85), 2.

106
This group was responsible for roughly a third of the publications in the official Reports from the Laboratory of the Royal College of Physicians, Edinburgh (vols. 1–7) between 1889 and 1895. On the laboratory, see SturdySteve, “Knowing Cases: Biomedicine in Edinburgh, 1887–1920,” Soc. Stud. Sci. 37 (2007): 659–89.

107
SymingtonJohnson, “Subsidised Scientific Books,” Brit. Med. J. 1 (1893): 267; The Edinburgh University Calendar 1889–90 (Edinburgh, 1889), 517.

108
SimpsonAlexander Russell, “Valedictory Address,” Trans. Edinburgh Obstet. Soc. 9 (1884): 1–8.PMC548518229612891

109
Jacyna, “‘A Host of Experienced Microscopists’” (n. 9), 249.10.1353/bhm.2001.007211423682

110
Barbour, Atlas (n. 85), 6.

111
LawrenceChristopher, “Incommunicable Knowledge: Science, Technology and the Clinical Art in Britain 1850–1914,” J. Contemp. Hist. 20 (1985): 503–20; and, for differences between the medical cultures of London and Edinburgh, see Lawrence, Rockefeller Money, the Laboratory, and Medicine in Edinburgh, 1919–1930 (Rochester, N.Y.: University of Rochester Press, 2005).10.1177/00220094850200040211617289

112
Trans. Obstet. Soc. London 29 (1887): 180.

113
BarnesRobertBarnesFancourt, A System of Obstetric Medicine and Surgery, Theoretical and Clinical for the Student and Practitioner, vol. 1 (London, 1884), 162.

114
BarnesRobert, A Clinical History of the Medical and Surgical Diseases of Women (London, 1873), 144–45.

115
“Congress of Obstetrics and Gynaecology at Brussels,” Lancet 140 (1892): 742–43, quotation on 743.

116
LockwoodCharles B., “An Address on the Teaching of Anatomy,” Brit. Med. J. 2 (1888): 1372–73.10.1136/bmj.2.1460.1372PMC219842220752526

117
MacalisterAlexander, “On Methods of Anatomical Study,” Brit. Med. J. 2 (1893): 879–83, quotations on 880–82. For other defenses of frozen sections, see Primrose, “Study of Anatomy” (n. 83); and “Discussion on the Teaching of Anatomy” (n. 48).

118
MacalisterAlexander, “Introductory Lecture on the Province of Anatomy,” Brit. Med. J. 2 (1883): 808–11. On Macalister and Cambridge anatomy, see Hurren, Dying for Victorian Medicine (n. 10), 175–217.10.1136/bmj.2.1191.808PMC237303320750741

119
Macalister, “Methods” (n. 117), 882.

120
See Cooke’s obituary in Lancet 153 (1899): 482; MortonL. T., “London’s Last Private Medical School,” J. Royal Soc. Med. 84 (1991): 682; Hurren, Dying for Victorian Medicine (n. 10), 97–101.

121
CookeThomas, A Plea for Practical Work in Anatomy (London, 1893), 4, 8.

122
CookeThomasCookeF. G. Hamilton, Tablets of Anatomy, Dissectional and Scientific (London, 1898), 152.

123
CookeThomas, “The Teaching of Anatomy,” Lancet 142 (1893): 1153–55, quotation on 1154.

124
CookeCooke, Tablets (n. 122), 152.

125
Lancet 142 (1893): 1215; Cooke, Plea (n. 121), 32.

126 *Brit. Med. J.* 2 (1898): 720; 1 (1899): 444–45; *Lancet* 153 (1899): 482.

127
Primrose, “Study of Anatomy” (n. 83); “Methods of Teaching Anatomy” (n. 43); Dublin J. Med. Sci. 107 (1899): 120–26.

128
CookeCooke, Tablets (n. 122), iv; Lancet 143 (1894): 162–63.

129
McGregor, “Rise and Development” (n. 93).

130
NewmanGeorge, Some Notes on Medical Education in England (London: HMSO, 1918), 21, 42; and Newman, Recent Advances (n. 92). Edinburgh anatomists and obstetricians took advantage of this new concern with “inductive” learning by collaborating on a groundbreaking series of stereoscopic atlases. The Edinburgh Stereoscopic Atlas of Anatomy (London: Caxton, 1905), edited by David Waterson, and Obstetrics (1908) depicted a range of anatomical specimens, including frozen and formalin-hardened sections, and living patients, allegedly providing “illustrations so pronounced, clear and definite, that the student can now study these stereograms with as much accuracy as if he had the actual preparation in his hands.” Quoted in SimpsonGeorge F. BarbourBurnetDavid, The Edinburgh Stereoscopic Atlas of Obstetrics (London: Caxton, 1908), vii.

131
Medical Education in London (n. 50), 157–58; BensleyRobert, “Department of Anatomy of the University of Chicago,” Meth. Prob. Med. Ed. 19 (1931): 34.

132
WatsonBenjamin P., “The Development of Obstetrics,” Can. Med. Assoc. J. 4 (1914): 469–80.20310497PMC406654

133
Sappol, Dream Anatomy (n. 4), 156; The Slice: Cutting to See (exhibition organized by Cabinet Magazine at the Architectural Association, London, 2010), http://cabinetmagazine.org/events/theslice.php.

134
DijckJosé van, The Transparent Body: A Cultural Analysis of Medical Imaging (Seattle: University of Washington Press, 2005), 118–37.

